# Achieving Chronic Care Equity by Leveraging the Telehealth Ecosystem (ACCTIVATE): A Multilevel Randomized Controlled Trial Protocol

**DOI:** 10.18103/mra.v12i11.6087

**Published:** 2024-11

**Authors:** Adenike Omomukuyo, Andy Ramirez, Aliyah Davis, Alexandra Velasquez, Adriana L Najmabadi, Marianna Kong, Rachel Willard-Grace, William Brown, Andrew Broderick, Karla Suomala, Charles E McCulloch, Nora Franco, Urmimala Sarkar, Courtney Lyles, Amber S Tran, Anjana E Sharma, Delphine S Tuot

**Affiliations:** 1 Department of Medicine, Division of Nephrology, Zuckerberg San Francisco General Hospital, University of California, San Francisco, San Francisco, CA, United States; 2 Department of Family & Community Medicine, University of California, San Francisco, School of Medicine, San Francisco, CA, United States; 3 Division of Prevention Science, Department of Medicine, University of California, San Francisco, San Francisco, CA, United States; 4 San Francisco Tech Council, San Francisco, CA, United States; 5 Department of Epidemiology and Biostatistics, University of California, San Francisco, San Francisco, CA, United States; 6 Zuckerberg San Francisco General Hospital Medical Library, University of California, San Francisco, San Francisco, CA, United States; 7 Division of General Internal Medicine, Zuckerberg San Francisco General Hospital, Department of Medicine, University of California San Francisco, San Francisco, CA, United States; 8 Action Research Center for Health Equity, Zuckerberg San Francisco General Hospital, University of California San Francisco, San Francisco, CA, United States; 9 UC Davis Center for Healthcare Policy and Research, UC Davis School of Medicine, University of California, Davis, Sacramento, CA, United States; 10 Health Informatics, Yale School of Public Health, New Haven, CT, United States

## Abstract

**Background:**

Racial/ethnic and socioeconomic disparities in diabetes and hypertension outcomes persist in the United States (U.S.), and worsened during the COVID-19 pandemic. This was in part due to suboptimal implementation of telehealth in U.S. safety-net settings alongside the pre-existing “digital divide” – structural determinants that limit access to digital tools by marginalized communities. To improve health equity, it is critical that health systems in the U.S. integrate principles of digital and health literacy for more equitable chronic disease care.

**Methods:**

We are conducting a 2x2 factorial randomized controlled trial, in partnership with a Community Advisory Board, assessing a multi-level intervention addressing barriers that affect the equitable use of telehealth amongst low-income patients in San Francisco County. Patient-level support is provided through the evidence-based strategies of health coaching and digital navigation (“digital coaching”); clinic-level support includes equity dashboards, patient advisory councils, and practice facilitation. We are randomizing 600 low-income, racially/ethnically diverse English and Spanish-speaking patients with uncontrolled diabetes to receive digital coaching (n=200) vs. usual care (n=400) for 3 months; and 11 public health primary care clinics to clinic support vs. usual care for 24 months. We aim to evaluate the impact of patient and clinic level interventions to determine individual effectiveness and potential synergistic impact on clinical and process measures related to diabetes and telehealth outcomes.

**Results:**

The study’s primary clinical outcome is change in patient-level Hemoglobin A1C (A1c); the primary process outcome is patient portal usage. Secondary clinical outcomes include changes in patient-level systolic blood pressure (SBP) and microalbuminuria (UACR), and changes in clinic-level A1c, SBP, and UACR. Secondary process outcomes assess patient-level changes in digital literacy, medication adherence, patient activation, and visit show rates, and clinic-level measures of telehealth adoption.

**Discussion:**

The ACCTiVATE trial tests a multi-level intervention developed through a stakeholder-engaged research approach and user-centered design to be feasible and acceptable for impacted communities. If efficacious, ACCTiVATE may provide a scalable model to improve chronic health outcomes and telehealth equity among marginalized racial/ethnic populations experiencing structural and interpersonal access barriers.

**Trial registration:**

ClinicalTrials.gov identifier NCT06598436. Registered 15 September 2024.

## Introduction

Diabetes (DM) and hypertension (HTN) cause significant cardiovascular (CV) burden that disproportionately impacts racially and ethnically minoritized populations and Americans with lower socioeconomic status (SES). Due to well-documented structural determinants of health, including barriers of access to health care,^1–3^ Black/African Americans are over twice as likely to die than White Americans from HTN^4^ and have the highest risk of death due to stroke.^5^ In the United States (U.S.), the risk of DM is 77% higher among Black/African Americans, and 66% higher among Latinx/Hispanic populations, compared to White Americans.^6,7^ For these high risk patients who receive care within safety-net healthcare systems, inadequate CV care delivery can also heighten susceptibilities to co-morbidities and complicate self-management.^8^

### IMPACT OF UNEVEN TELEHEALTH IMPLEMENTATION AND THE DIGITAL DIVIDE ON PATIENT HEALTH OUTCOMES

The COVID-19 pandemic resulted in decreased access to health care and under-treatment of chronic conditions among minoritized populations in the United States.^9–13^ The explosive expansion of telehealth-based care in the U.S. (including synchronous telemedicine visits on phone and audio, and asynchronous care via patient portals and remote patient monitoring)^14^ was unevenly implemented nationwide.^15^ Most publicly funded safety-net clinics predominantly offer telemedicine phone visits (vs. video), default to in-person visits despite patients’ preferences, have fewer established workflows leveraging health information technology (IT), and provide suboptimal patient support for portal communication.^15^ Providers in these clinics, who offer services for a high proportion of low-income patients,^16^ face challenges with leveraging high-quality tools in the digital ecosystem to manage patients’ chronic health conditions.^17–19^

Existing structural barriers already limit telehealth availability for individuals with low income and educational attainment, older adults, and those with limited English proficiency, thus creating a “digital divide”.^20,21^ These include socioeconomic barriers to device access, limited educational opportunities resulting in low health literacy and digital literacy, “digital redlining” impeding internet access, and lack of contextually-tailored programming to support diverse patients to participate in digital visit modalities.^22–26^ The confluence of health system challenges with implementing telehealth and the digital divide risks further exacerbating socioeconomic and racial/ethnic disparities in DM and HTN burden and outcomes.

### PRACTICE FACILITATION, DIGITAL NAVIGATORS, AND HEALTH IT ADOPTION

Safety-net practices face numerous clinic-level barriers that limit the use of telehealth for care delivery. These include care team biases in offering telehealth modalities to marginalized patients, lack of support to help patients engage with the technology, and suboptimal data about health IT use.^27–29^ Practice facilitation has been proven to be an effective strategy for transforming primary care clinics into patient-centered medical homes, enhancing equitable care delivery, and increasing health IT adoption.^30,31^ It entails in-depth assessment of current practices; care team training in cultural humility and shared decision-making; measuring and reviewing data stratified by race/ethnicity; incorporating feedback from clinic stakeholders to design patient-centered workflows; and guiding quality improvement projects to accomplish clinic equity goals.^32^ Practice facilitation has also supported the establishment of patient and community advisory boards^33,34^ which can improve clinics’ responsiveness to community needs that impact equity.^35^ Given its known efficacy, practice facilitation with community engagement holds potential to enhance use of telehealth to reduce disparities in chronic disease outcomes, but evidence is lacking regarding its use for tackling the digital divide.

On the individual level, digital navigation and health coaching are promising approaches to address barriers in telehealth care access. Digital navigators, defined as non-clinical staff who support patients to access and utilize digital health tools,^36^ can guide patients to community resources to access devices or sites with internet access^37^ and provide direct patient support to improve digital knowledge, skills, and confidence in using health IT.^36^ Similarly, health coaching performed in-person, via telephone or through text-messaging to augment in-person chronic disease care^38–41^ improves clinical indicators such as Hemoglobin A1c (A1c),^42^ medication adherence,^43–45^ and patient experience^42,46,47^ with maintenance of benefit up to at least one year.^48^ Community health workers, who can provide health coaching, have thus been embedded into Medicaid and other Center for Medicare and Medicaid Innovation reimbursement policy changes^49^ signaling a potential paradigm shift among safety-net practices who may consider implementing health coaching strategies. The role of health coaching to increase telehealth for virtual chronic disease care has not yet been explored, and little evidence has linked digital literacy support to virtual chronic disease self-management, which is key to improving chronic health outcomes.

### ACHIEVING CHRONIC CARE EQUITY BY LEVERAGING THE TELEHEALTH ECOSYSTEM

To mitigate these translational gaps, we describe the design and protocol of a randomized controlled trial entitled “Achieving Chronic Care equiTy by leVerAging the Telehealth Ecosystem” (ACCTiVATE). This study assesses the impact of a multi-level intervention addressing patient and clinic-level challenges to leverage health IT for chronic health conditions, while evaluating its effectiveness, integration into clinical care, and potential for spread to systems serving low-income, diverse patients. The design and protocol were developed with community and stakeholder input throughout, which is an approach that is recommended for enhancing acceptability and efficacy for impacted populations.^50^ We hypothesize that the use of efficacious support strategies for patients and clinical teams will improve glycemic control among racial/ethnic subgroups through increased patient digital literacy, medication adherence, and technology-enabled enhanced care access.

## Methods

### CONCEPTUAL FRAMEWORK

The intervention utilizes core elements from the National Institute on Minority Health and Health Disparities (NIMHD) Research Framework,^51^ adapted for digital health equity.^52^ This framework acknowledges multiple levels of systemic, institutional, community, and individual barriers that impact health equity with regards to telehealth access, including digital redlining, bias in healthcare interactions, and decreased access to digital skills. To address these barriers, the ACCTiVATE study consists of: (1) a patient-level intervention that is a short-term, linguistically concordant, contextually tailored digital coaching program, which aims to improve digital literacy^36^ and engages patients in goal-setting and shared decision-making^53^; and (2) a clinic-level intervention, including practice facilitation strengthened by patient/community advisory boards and telehealth equity dashboards.

#### Stakeholder Engagement

The design of ACCTiVATE was developed with community and stakeholder involvement. Study co-investigators presented telehealth access disparities data to an existing COVID-19 research patient and community advisory board in November of 2020,^54^ and formulated the interventional approach with their recommendations, along with suggestions from two patient advisory councils at a family medicine clinic from 2020-2021. Details of the digital navigation interventional components have been developed in partnership with the San Francisco Tech Council, a community-based organization committed to addressing digital disparities^55^ that is an official co-investigator on the study team. The study is being performed with an ACCTiVATE-specific multidisciplinary community advisory board (CAB), including members of the public library network, local civic leadership, English and Spanish-speaking patients, primary care team staff, and telehealth operational leaders. The CAB meets quarterly. They are integral to the intervention component refinement, utilizing a user-centered design approach,^56,57^ reviewing study instruments and protocols, providing guidance on study barriers, and informing the interpretation and dissemination of results; members are reimbursed with gift cards for their time.

#### Study Design

ACCTiVATE is a prospective, non-blinded, 2x2 factorial randomized controlled trial with two levels of randomization (Figure 2). Half of the 11 participating clinics are randomized to receive practice facilitation (n=5) or usual care (n=6) for 24 months. Within each clinic, we are recruiting/randomizing eligible patients in a 1:2 ratio to receive tailored digital coaching (n=200) or usual care (n=400) for 3 months. Given the incremental personnel and opportunity costs of implementing a digital navigation program, this study design with 4 study arms allows for in-depth understanding of each intervention level’s individual and synergistic impact.

### STUDY POPULATION AND SETTING

The study is taking place in the San Francisco Health Network (SFHN), a public health network of primary and specialty care clinics. SFHN serves the low-income population of San Francisco, with high proportions of racial/ethnic minorities; 39.1% are Hispanic/Latinx, 25.3% are Asian, and 13.9% are African American. Most SFHN patients are publicly insured or uninsured (covered through a county-specific insurance if ineligible for Medicaid or Medicare) and 19.1% have limited English proficiency.^58^

### INCLUSION/EXCLUSION SUMMARY

#### Primary care clinics:

Eleven primary care federally qualified health centers that are part of the SFHN are participating in this study. All clinics in the network care for a racially, ethnically, and linguistically diverse patient population with a high prevalence of DM and other co-morbid conditions.

#### Patients:

Eligible patients include English or Spanish-speaking adults aged 18 and older with at least 1 visit at a participating SFHN primary care site in the last 24 months, who have diabetes with a last A1c ≥ 8.0%. Patients with higher-than-average digital literacy are excluded from this study as they may not benefit from a digital coaching intervention.^59^ Other exclusion criteria include patients with end-stage or terminal conditions that would make it inappropriate to focus on chronic disease management, cognitive impairments defined by the inability to restate study goals during the verbal consent process, and lack of any phone access. Pregnant people are also excluded as their care plans may involve unique blood pressure or glucose goals and management strategies.

### RECRUITMENT AND RANDOMIZATION

#### Clinic-Level Intervention

We are enrolling 11 clinics from the 14 SFHN primary care sites. Randomization occurred by clinic, as clinic-level workflows and structures directly impact adoption of telehealth workflows. Prior work by our team and others has demonstrated that all members of the primary care team are needed for practice transformation, particularly those aimed at combating inherent bias and incorporating new technology into clinical care delivery. This level of randomization leverages this team-based approach to chronic care delivery and minimizes potential for contamination among providers.

#### Patient-Level Intervention

ACCTiVATE study team members are recruiting based on electronic health record (EHR) data identifying primary care-enrolled patients with diabetes meeting inclusion criteria. After receiving assent from primary care providers to approach their patients, study team members offer study enrollment and obtain consent using a script that has been developed to address well-known barriers to study participation among racial/ethnic minorities and individuals of low socioeconomic status, including mistrust of research endeavors, economic and time constraints, transportation difficulties, and disease burden.^60–62^ Enrollment and onboarding protocols are developed in partnership with the study CAB. All participants receive $50 at the beginning of the study and at each additional study visit for a total of $200 per participant.

We employ a stratified randomization strategy. Clinics are randomized to assure balance by race/ethnicity; individual participants are randomized within each clinic. Randomization was conducted by a research statistician who has not made prior contact with participants, enabling blinded randomization with allocation concealment. The randomization scheme ensures that all study arms have equivalent proportions of Black and Latinx participants, as denoted in the EHR.

### INTERVENTIONS

#### Clinic-Level Intervention Arms

**Figure 1:**
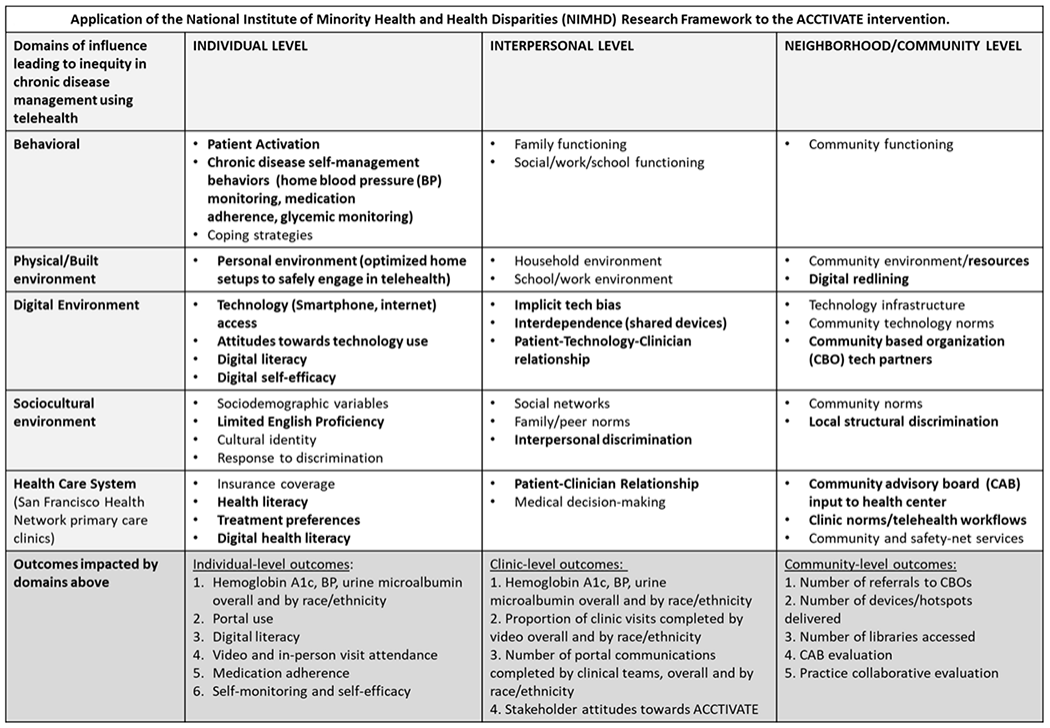
Multilevel framework of digital equity informed by the National Institute on Minority Health and Health Disparities (NIMHD) conceptual model. Bolded elements are those directly impacted by ACCTiVATE.

**Figure 2:**
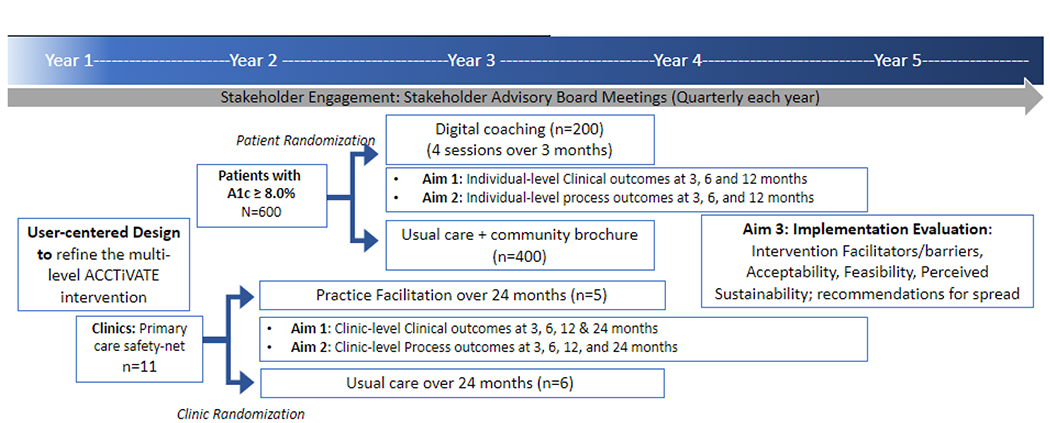
Study Design, Outcomes, and Timeline

1. **Practice Facilitation Arm:** Our clinic-level Practice Facilitation is adapted from the successful primary care practice transformation model employed by the UCSF Center for Excellence in Primary Care^63^ and focuses on the interpersonal and community levels of influence depicted in our conceptual model (Figure 1). It includes the following components:
  - *Implementation of practice trainings* with designated telehealth equity champions from each clinic, with prospective inclusion of medical leadership, primary care clinicians, pharmacists, front desk clerks, and medical assistants. Trainings include education regarding the digital divide and patient chronic disease self-management, review of diabetes management quality goals, and linkages with network-wide diabetes equity initiatives such as panel management, greater use of continuous glucose monitoring, and healthy food voucher programs.^64^
  - *Establishment of patient/community advisory councils (PACs)* at each clinic, led in accordance with best practices.^78^ These PACs are recruited from active patients at each clinic and local community leaders with experience related to telehealth access. PACs meet regularly with the goal of enhancing clinic-level understanding of interpersonal and community barriers to telehealth engagement, and providing priority setting and guidance to clinic quality improvement goals and projects.
  - *Dissemination of telehealth equity dashboards to staff* that depict clinic-level portal enrollment and telemedicine visit rates, stratified by race/ethnicity and language. Participating clinics review these dashboards and discuss provider and clinic-level norms and biases that may contribute to differences in telehealth use. Digital disparities are targeted with specific quality improvement projects, developed by telehealth equity champions applying LEAN methodology^65–67^ in coordination with clinic PACs.
2. **Usual Care Arm:** Clinics randomized to usual care are not supported by Practice Facilitation domains. Clinic leaders and providers have access to the telehealth equity dashboards through the EHR and other existing network-level resources relevant to diabetes and telehealth.

#### Patient-Level Intervention Arms

**Figure 3.**
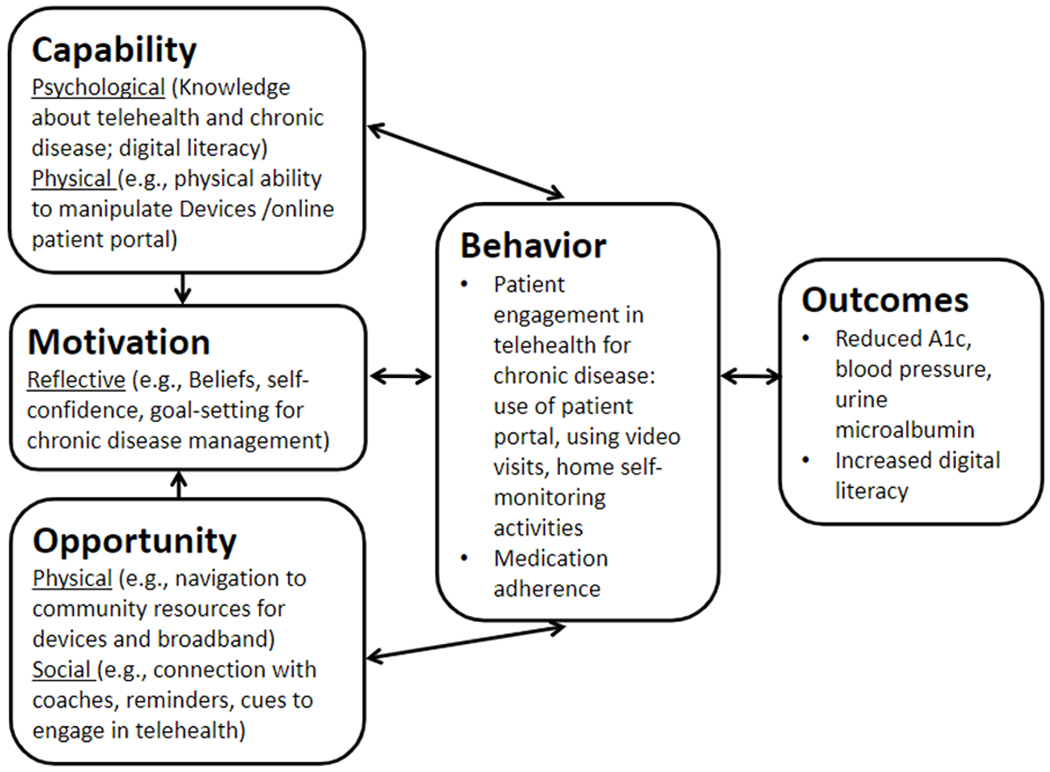
Application of the Capability, Opportunity, Motivation – Behavior (COM-B) Theoretical Model to the ACCTiVATE Patient-facing Intervention

1. **Intervention Arm: Digital Coaching Program:** The mechanism of the ACCTiVATE patient intervention is grounded in the Capability, Opportunity, Motivation-Behavior (COM-B) model, an evidence-based theory of individual behavioral change (Figure 3).^68^ Patients, particularly those who are low-income with limited digital literacy, currently have inadequate support to access the knowledge, skills, and confidence necessary to engage in telehealth. The ACCTiVATE digital coaching program improves capability by building skills in self-monitoring and navigation of a patient portal and a video telemedicine encounter. It also improves physical opportunity to engage in telehealth by providing navigation to community-based organizations or city-funded sites to access broadband or video enabled smartphones.
  The curriculum is co-developed with the Community Advisory Board through iterative meetings. Board members review the drafted curriculum (Table 2) and participate in role-playing activities to ensure the content is feasible and acceptable to SFHN populations, specifically those experiencing barriers in accessing telehealth.
  ACCTiVATE digital coaches conduct sessions with intervention arm patients over 3 months. Coaches connect with participants using their preferred mode of communication; this may be in-person in the community or at a clinical site, over the phone, and/or via other modalities such as text message and video chat based on patient preference.
  ACCTiVATE digital coaches enhance capability and opportunity by liaising patients with local community resources available for low-cost or free devices, internet access, and digital skills classes. The digital coaches can also utilize the web-based, interactive patient portal training platform entitled “YourChart,” resulting from a co-design process that was led by the co-investigators of San Francisco Tech Council.^55^ YourChart simulates the experience of navigating the online patient portal without a login or activated account, and is operational in English and Spanish. YourChart can allow participants to acquire skills and build confidence through practice without compromising actual patient health data, and includes learning mockups for scheduling appointments, messaging providers, reviewing test results, and refilling prescriptions. When delivered by ACCTiVATE digital coaches, the YourChart training can be customized to the motivations and goals of individual patients to reinforce the benefits of patient portal use, in addition to promoting greater adoption.
  Coaches provide motivation through goal-setting and reinforcing the positive impacts of engaging with health IT and improving overall health. They also provide culturally responsive support with an equity lens, highlighting how social/cultural contexts and norms, individual responses to discrimination, and technology bias may have impacted prior telehealth engagement.
  These technical and behavioral supports may lead to enhanced self-monitoring, portal engagement, telemedicine visit participation, and medication adherence, which in turn can improve chronic health conditions.
2. **Usual Care Arm:** Patients randomized to the usual care arm receive a brochure that encourages participation in a digital training and portal enrollment session offered by the public hospital’s library. These training and enrollment sessions are available to all SFHN patients on campus and online.

### DATA COLLECTION

ACCTiVATE participants take part in a baseline visit, where socio-demographic data (age, sex, zip code, income, educational attainment), co-morbid conditions, and baseline measures of digital literacy, medication adherence, patient activation, and home monitoring are ascertained. Follow-up visits occur by telephone or video at 3, 6, and 12 months during which the study team measures digital literacy, medication adherence, patient activation, and home glucose and BP monitoring. Other process outcomes and clinical outcomes are ascertained from the EHR. See Tables 3 and 4 for a detailed summary of study outcomes.

### IMPLEMENTATION ASSESSMENT

In addition to the primary trial procedures outlined above, we plan to conduct a mixed methods evaluation of implementation outcomes in the ACCTiVATE study; this will be described in future papers.

## Results

### OUTCOMES

#### Clinical Outcome Measures

The primary clinical outcome is patient-level change in A1c. Secondary clinical outcomes include: patient-level change in systolic blood pressure (SBP) and microalbuminuria (UACR), and clinic-level change in A1c, SBP, and UACR. All participant measures of A1c, SBP, and microalbuminuria (UACR) from one year prior to study initiation through 12 months after implementation are pulled from the EHR. We examine changes in individual patient A1c, SBP, and UACR from baseline to 3 months, 6 months (primary outcome), and 12 months. Clinic-wide measures of A1c, SBP, and UACR are similarly extracted from the EHR, and ascertained at baseline and 3, 6, 12, and 24 months. We use measures closest to each time point +/− 2 months. In accordance with standing order protocols, A1c, SBP, and UACR are captured regularly for clinical care among individuals with DM.

#### Process Outcome Measures

The primary process outcome is change in patient portal use. Secondary process outcomes explore patient-level digital literacy, self-reported medication adherence, patient activation, and visit show rates. An additional secondary process measure is the proportion of clinic visits completed by video. All process outcomes are ascertained by a research data analyst blinded to randomization. Patient-level outcomes are obtained at baseline and at months 3, 6, and 12. Clinic-level process measures are ascertained from the EHR at baseline and months 3, 6, 12, and 24.

##### Patient Portal Use.

Using the EHR, we calculate the total number of EHR portal logins per patient over each 3-month period.^72^

##### Digital Literacy.

Digital Literacy is ascertained with the Digital Equity Screening Tool (DEST).^69^ The DEST is a self-reported tool that assesses patient experience with technology. It includes 5-items: Device access, Internet access, Digital literacy, Digital assistance, and Language barriers.

##### Self-reported Medication Adherence.

We use the eight-item Morisky Medication Adherence Scale (MMAS-8)^70^ that has been validated in low-income and Spanish-speaking populations and is associated with HTN control.^73,74^

##### Patient Activation.

Patient activation is measured by the Patient Activation Measure (PAM).^71^ Patients answer using a Likert scale of answers ranging from 1-5, with 1 signifying “Almost Never” and 5 signifying “Almost Always”.

##### Visit Show Rates.

Through use of the EHR, we obtain participant quarterly visit show rate (# completed encounters/total scheduled ambulatory visits) overall and for telehealth video visits. We conduct periodic validity checks of the EHR data with chart review.

##### Clinic-level Video Visit Rates.

Through the EHR, we capture the proportion of all ambulatory clinic visits that are telehealth video visits on a quarterly basis.

### ANALYSIS

We are performing an intention-to-treat analysis, assessing impact of the patient-facing intervention and the clinic-facing intervention on clinical and process outcomes, each versus usual care. We include random effects for both clinic and participants to account for clustered and repeated measures. Because patients see multiple providers within the same clinic, the clinic random effect should suffice to control provider differences. The primary analyses use linear mixed models to assess changes in A1c compared to baseline between study arms at 3, 6 (primary outcome), and 12 months by testing each of the times by intervention interactions. To improve precision and accommodate any unbalanced randomization, all analyses are adjusted for age, Charlson co-morbidity index, and baseline patient activation. We assess for an interaction between the effects of the clinic-level and patient-level interventions on A1c. If an interaction is identified (p<0.05), we then determine synergy or lack thereof and conduct stratified analyses by study arm. For all outcomes, p-values are two-sided, and p-values <0.05 are considered significant. We conduct subgroup analyses among minoritized (Black and Latinx) populations, within each of the Black and Latinx populations, and among those with higher/lower digital literacy. We also conduct interaction analyses and consider subgroup analyses by sex.

While our analyses for secondary clinical outcomes and process outcomes follow suit, some measures may not be approximately normally distributed. When residuals appear non-normally distributed, we use transformations to make the data more normally distributed or, failing that, use bootstrapping to avoid parametric assumptions.

### SAMPLE SIZE CONSIDERATIONS

Sample size and power calculations were performed for both primary clinical and process outcomes of change in patient-level A1c and patient portal use. Baseline data from the potentially eligible study population suggests a baseline of A1c of 10.0% (SD 1.8) and an intraclass correlation of 0.18 across primary care clinics. We have 80% power to detect a 0.4% difference in mean A1c among participants randomized to digital coaching vs. usual care and 1.3% difference among those whose clinics are randomized to practice facilitation vs. usual care. Baseline data for patient portal use indicates that 38.4% (SD 0.49) of eligible patients have portal access. We have 80% power to detect a 12% difference in patient portal access among those randomized to digital coaching vs. usual care and a 22% difference among those whose clinics are randomized to practice facilitation vs. usual care. Since portal messaging in our system is quite low, any increase is meaningful for stakeholders.

## Discussion

The current status quo of telehealth-based chronic disease care in safety net care settings in the United States is ad-hoc, inequitable by race/ethnicity and language, lacks patient support for digital access needs, and is predominantly reliant on telephone encounters. This is largely due to the impact of the COVID-19 pandemic on already stressed public health care delivery systems, limiting their capacity to proactively address disparities in digital technology use.^75,76^

Direct patient support via digital coaching can meet the needs of patients who have been left behind in the digital divide, including those with reduced digital literacy and limited access to smartphones and broadband, thus increasing their confidence in using digital technologies and engaging in virtual care. Since health coaches and digital navigators have both been found to be beneficial, the ACCTiVATE intervention proposes a novel “digital coaching” program that combines the two to enhance patient use of health IT. Furthermore, the ACCTiVATE digital coaches provide chronic disease support and increase access to telehealth by bridging the silos of healthcare resources and digital supports from community-based organizations. With the potential to receive reimbursement for digital coaching activities through new community health worker insurance policies,^77^ future adaptation of the ACCTiVATE digital coaching program is also likely to be financially sustainable.

Primary care clinic support through practice facilitation can empower team members to address racial/ethnic disparities in telehealth use through more equitable screening/offering of digital technologies, resources to prepare patients for virtual chronic disease management, and consistent review of telehealth equity data. Practice coaching has been studied for quality improvement and practice transformation,^78^ but has not been implemented in a targeted strategy to address health disparities and the digital divide. ACCTiVATE deploys the evidence-based strategy of practice facilitation with a novel focus on telehealth equity to improve chronic disease care delivery in safety-net settings.

The ACCTiVATE trial tests a multi-level intervention developed through a stakeholder-engaged research approach and user-centered design, in order to be most feasible and acceptable for impacted communities. If efficacious, ACCTiVATE may provide a scalable model to improve chronic health outcomes among populations experiencing racial/ethnic marginalization, and support increased telehealth equity by addressing multiple levels of structural and interpersonal access barriers.

## Figures and Tables

**Table 2:** ACCTiVATE core curricular components. Sessions will involve various themes as directed by participant goals.

ACCTIVATE Patient Curriculum Themes	Behavior change goal mapped to COM-B^68^	Health Information Technology/Digital navigation curriculum	Chronic disease health coaching curriculum
Getting Started	Address physical/digital **capability, motivation, and opportunity** to engage in telehealth for chronic disease care	-Review digital literacy level and current device use -Support digital device and broadband access by referrals to community organizations/resources -Assure access to glucometer, continuous glucose monitoring, and/or home BP cuff and glucometer (if appropriate) -Enrollment in online patient portal -Train/practice to enroll in video visits	-Establish personal chronic care goals -Assess and build on patient knowledge of DM, HTN, and/or CKD terms and goals (“Ask-Tell-Ask”) -Train in home glucose measurement or assessment, home BP measurement using devices (“Teach Back” to ensure accurate use)
Preparing for a telemedicine visit	Address psychological **capability** and **motivation** to participate in telehealth chronic disease management	-“What can my portal do for my health?” -“ What can a telemedicine visit do for my health?” -Practice skills in portal login and navigation for telemedicine video visit -Practice messaging and securing appointments or refills via portal	-Build skills in visit preparation: e.g., identifying questions for visit -Review chronic care medications, elicit understanding and current barriers to adherence, goal-setting for medication adherence
Closing the loop with telehealth	Review new skills/successful outcomes and **motivate** to continue	-Discuss successes and challenges of telemedicine encounter; troubleshoot as needed -Review goals and close loop after a visit with the “teach back” method -Train/practice using the patient portal to send messages, secure appointments, and refills -Refer to community resources for additional digital skill classes, trainings, and workshops	-“Teach back” to reinforce basics of DM, HTN and/or CKD and goals - Assess medication adherence and address barriers -Create action plan for future telehealth chronic care actions (e.g., use of portal to send message or set up appointment)
Reinforcing Your Skills	**Reinforce** maintenance of digital and chronic disease self-management behaviors	-Discuss facilitators and barriers to prior portal messages and telemedicine encounters -Teach-back in navigating video visits and patient portal -Review eligibility for other community resources	- “Teach back” on new chronic disease management information -Create action plan for personal chronic care self-management goal (e.g., nutrition, physical activity)

**Table 3:** Clinical Outcomes

Primary or Secondary	Outcome Measure	Method of Ascertainment and Definition	Timepoints
Primary	Patient-level Hemoglobin A1C (%)	Hemoglobin A1C values from EHR Change in A1C (%) is determined by subtracting month 3, 6, and 12 A1C values from baseline A1C.	Baseline, 3-mo, 6-mo, and 12-mo
Secondary	Patient-level Systolic BP (mmHg)	Clinic-based BP readings from EHR Change in SBP (mmHg) is determined by subtracting month 3, 6, and 12 SBP values from baseline SBP.	Baseline, 3-mo, 6-mo, and 12-mo
Secondary	Patient-level Microalbuminuria UACR (mg/g)	Microalbuminuria values from EHR Change in microalbuminuria (mg/g) is determined by subtracting month 3, 6, and 12 microalbuminuria values from baseline microalbuminuria.	Baseline, 3-mo, 6-mo, and 12-mo
Secondary	Clinic-level Hemoglobin A1C (%)	Hemoglobin A1C values from EHR Clinic-wide averages for month 3, 6, 12, and 24 A1C values are subtracted from clinic-wide average baseline A1C to determine the change in clinic-wide A1C (%).	Baseline, 3-mo, 6-mo, 12-mo, and 24-mo
Secondary	Clinic-level Systolic BP (mmHg)	BP readings will be obtained from the EHR Change in clinic-wide SBP (mmHg) is determined by subtracting average clinic-wide SBP for month 3, 6, 12, and 24 from baseline clinic-wide average SBP (mmHg).	Baseline, 3-mo, 6-mo, 12-mo, and 24-mo
Secondary	Clinic-level Microalbuminuria UACR (mg/g)	Microalbuminuria values from EHR Clinic-wide averages for month 3, 6, 12, and 24 UACR values are subtracted from clinic-wide average baseline UACR to determine the change in clinic-wide UACR (mg/g).	Baseline, 3-mo, 6-mo, 12-mo, and 24-mo

**Table 4:** Process Outcomes

Primary or Secondary	Outcome Measure	Method of Ascertainment	Timepoints
**Primary**	Patient Portal Use	Electronic Health Record (EHR)	Baseline, 3-mo, 6-mo, and 12-mo
**Secondary**	Digital Literacy	Digital Equity Screening Tool (DEST)^69^	Baseline, 3-mo, 6-mo, and 12-mo
**Secondary**	Medication Adherence (Patient Reported)	Morisky Medication Adherence Tool (MMAS-8)^70^	Baseline, 3-mo, 6-mo, and 12-mo
**Secondary**	Patient Activation	Patient Activation Measure PAM)^71^	Baseline, 3-mo, 6-mo, and 12-mo
**Secondary**	Visit Show Rates	Electronic Health Record (EHR)	Baseline, 3-mo, 6-mo, and 12-mo
**Secondary**	Proportion of Clinic Visits Completed by Video	Electronic Health Record (EHR)	Baseline, 3-mo, 6-mo, 12-mo, and 24-mo
